# Activity of Epsilon-poly-L-lysine against Multidrug-Resistant *Pseudomonas aeruginosa* and *Klebsiella pneumoniae* Isolates of Urinary Tract Infections

**DOI:** 10.3390/biomedicines12030638

**Published:** 2024-03-13

**Authors:** Telma de Sousa, Carolina Sabença, Miguel Ribeiro, Mario Pino-Hurtado, Carmen Torres, Michel Hébraud, Olimpia Alves, Sara Sousa, Eliana Costa, Gilberto Igrejas, Patrícia Poeta

**Affiliations:** 1 MicroART-Antibiotic Resistance Team, Department of Veterinary Sciences, University of Trás-os-Montes and Alto Douro, 5000-801 Vila Real, Portugal; telmas@utad.pt (T.d.S.); anacarolina@utad.pt (C.S.); 2 Department of Genetics and Biotechnology, University of Trás-os-Montes and Alto Douro, 5000-801 Vila Real, Portugal; jmribeiro@utad.pt (M.R.); gigrejas@utad.pt (G.I.); 3 Functional Genomics and Proteomics Unit, University of Trás-os-Montes and Alto Douro, 5000-801 Vila Real, Portugal; 4 Associated Laboratory for Green Chemistry, University NOVA of Lisbon, 1099-085 Caparica, Portugal; 5 CQ-VR, Chemistry Research Centre, Food and Wine Chemistry Laboratory, University of Trás-os-Montes and Alto Douro, 5000-801 Vila Real, Portugal; 6 Area of Biochemistry and Molecular Biology, University of La Rioja, 26006 Logroño, Spain; mario-sergio.pino@unirioja.es (M.P.-H.); carmen.torres@unirioja.es (C.T.); 7 Institut National de Recherche Pour l’Agriculture, l’Alimentation et l’Environnement (INRAE), Université Clermont Auvergne (UCA), UMR Microbiologie Environnement Digestif Santé (MEDiS), 63122 Saint-Genès-Champanelle, France; michel.hebraud@inrae.fr; 8 Clinical Pathology Department, Hospital Centre of Trás-os-Montes and Alto Douro, 5000-508 Vila Real, Portugal; orvalves@chtmad.min-saude.pt (O.A.); sisousa@chtmad.min-saude.pt (S.S.); ecsvalente@chtmad.min-saude.pt (E.C.); 9 CECAV—Veterinary and Animal Research Centre, University of Trás-os-Montes and Alto Douro, 5000-801 Vila Real, Portugal; 10 Veterinary and Animal Research Centre, Associate Laboratory for Animal and Veterinary Science (AL4AnimalS), 5000-801 Vila Real, Portugal

**Keywords:** *Pseudomonas aeruginosa*, *Klebsiella pneumoniae*, antibiotic resistance, biofilm, Epsilon-poly-L-lysine

## Abstract

*Pseudomonas aeruginosa* and *Klebsiella pneumoniae* are notorious for their resistance to antibiotics and propensity for biofilm formation, posing significant threats to human health. Epsilon-poly-L-lysine (ε-PL) emerges as a naturally occurring antimicrobial poly(amino acid), which positions it as a prospective agent for addressing challenges linked to multidrug resistance. ε-PL symbolizes a promising avenue in the pursuit of efficacious therapeutic strategies and warrants earnest consideration within the realm of clinical treatment. Thus, our objective was to determine the antibiotic susceptibility profiles of 38 selected *P. aeruginosa* and ESBL-producing *K. pneumoniae* clinical isolates and determine the ability of ε-PL to inhibit biofilm formation. After PCR analysis, detection of genes related to β-lactamases was observed among the selected isolates of *P. aeruginosa* [*bla*_SPM_ (35.7%), *bla*_KPC_ (35.7%), *bla*_SHV_ (14.3%), *bla*_CTX-M_ (14.3%), *bla*_OXA_ (14.3%), *bla*_TEM_ (7.1%), *bla*_PER_ (7.1%), *bla*_VIM_ (7.1%), and *bla*_VIM-2_ (7.1%)] and *K. pneumoniae* [*bla*_CTX-M_ (91.7%), *bla*_TEM_ (83.3%), *bla*_KPC_ (16.7%), *bla*_NDM_ (12.5%), and *bla*_OXA_ (4.2%)]. The results of testing the activity of ε-PL against the clinical isolates showed relatively high minimum inhibitory concentrations (MICs) for the *P. aeruginosa* (range: 8–64 µg/mL) and *K. pneumoniae* isolates (range: 16–32 µg/mL). These results suggest the need for prior optimization of ε-PL concerning its viability as an alternative to antibiotics for treating infections caused by *P. aeruginosa* and *K. pneumoniae* of clinical origin. It is noteworthy that, in the context of a low antibiotic discovery rate, ε-PL could play a significant role in this quest, considering its low toxicity and the unlikely development of resistance. Upon exposure to ε-PL, *P. aeruginosa* and *K. pneumoniae* isolates exhibited a reduction in biofilm production, with ε-PL concentration showing an inverse relationship, particularly in isolates initially characterized as strong or moderate producers, indicating its potential as a natural antimicrobial agent with further research needed to elucidate optimal concentrations and application methods across different bacterial species. Further research is needed to optimize its use and explore its potential in various applications.

## 1. Introduction

*Pseudomonas aeruginosa* and *Klebsiella pneumoniae* are opportunistic gram-negative pathogens that can colonize and cause human infections [1]. These pathogens are one of the most common causes of nosocomial infections and are frequently responsible for a variety of healthcare-related infections, including pneumonia, bloodstream, meningitis, urine, and wound or surgical site infections, among others [2,3,4,5,6]. *P. aeruginosa* and *K. pneumoniae* can potentially develop resistance to many classes of antibiotics, such as carbapenems, and treating infections with these bacteria can be very challenging [7,8,9]. Multidrug-resistant (MDR) and extensively drug-resistant (XDR) Gram-negative bacteria are major public health problems worldwide. The main difference between these two bacteria is that *P. aeruginosa* is a glucose-non-fermenting and oxidase-positive bacteria, and *K. pneumoniae* is a glucose-fermenting and oxidase-negative bacteria. Also, another difference is their type of respiration, namely that *K. pneumoniae* is facultatively anaerobic as opposed to *P. aeruginosa*, which is considered an aerobic bacteria. Consequently, biofilm production and formation have different metabolic processes [10,11]. Biofilm formation is a highly conserved mechanism of bacterial adaptation and is associated with developing resistance to many antibiotics [12]. Thus, to combat multidrug-resistant *P. aeruginosa* and *K. pneumoniae*, alternative treatment strategies for reducing MDR bacteria have become a prominent research topic.

ε-PL is a hydrophilic cationic linear homo-poly-amino acid, generally composed of 25 to 35 identical L-lysine residues with a unique structure characterized by the peptidic bonds between the ε-amino and α-carboxy groups [13]. Within the scope of research on ε-PL, new modification strategies stand out for obtaining structures beyond linear configurations, encompassing dendritic, hyperbranched, and functionalized forms of ε-PL. These variations aim to enhance specific characteristics, broaden applications, and meet diverse needs [13]. ε-PL can inhibit various microorganisms, such as most bacteria [14], and it is “Generally Recognized As Safe” (GRAS) as a food preservative. The antimicrobial activity of ε-PL is closely related to the number of repetitive L-lysine residues. It has been shown that more than 10 residues are required for ε-PL to exert adequate antibacterial activity. It has already been demonstrated that α-PL is a much less potent antimicrobial compound than ε-PL [15]. Studies testing the antimicrobial capacity of this peptide have shown that it can be electrostatically adsorbed to the surface of the bacteria, followed by the removal of the outer membrane. Consequently, this results in the abnormal distribution of the cytoplasm, causing damage to the bacteria [16].

This study sought to investigate a group of specifically chosen *P. aeruginosa* and ESBL-producing *K. pneumoniae* isolates sourced from urine samples. Our focus was to explore the capability of these strains in forming biofilms and to assess the antimicrobial activity of ε-PL against these pathogens.

## 2. Materials and Methods

### 2.1. Samples and Bacterial Strains

Thirty-eight selected *P. aeruginosa* (*n* = 14) and ESBL-producing *K. pneumoniae* (*n* = 24) isolates recovered from urine samples at the Medical Centre of Trás-os-Montes and Alto Douro between September 2021 and June 2022 were included in this study. These strains were identified using VITEK 2^®^ COMPACT (bioMérieux, Auvergne-Rhône-Alpes, France), and their identification was confirmed in the medical microbiology laboratory by seeding *P. aeruginosa* and *K. pneumoniae* isolates on *Pseudomonas* Agar Base supplemented with CN (Liofilchem, Rosetodegli, Abruzzi, Italy) medium and HiCrome *Klebsiella* Selective Agar Base medium (HiMedia Laboratories, Maharashtra, India), respectively. Both pathogens were incubated at 37 °C for 24–48 h. The isolates were cryopreserved at −20 °C in skim milk.

### 2.2. Antimicrobial Susceptibility Testing

The phenotypic resistance characterization of the isolates was performed by the Kirby–Bauer disk diffusion method following EUCAST standards (2022) [17]. Twelve antibiotics were tested for *P. aeruginosa* (charge on disks): piperacillin (30 μg), ticarcillin-clavulanic acid (75–10 μg), ceftazidime (10 μg), cefepime (30 μg), aztreonam (30 μg), imipenem (10 μg), doripenem (10 μg), meropenem (10 μg), amikacin (30 μg), tobramycin (10 μg), gentamicin (10 μg), and ciprofloxacin (5 μg). The minimum inhibitory concentration (MIC) by microdilution method was performed for piperacillin-tazobactam antibiotics (resistant when MIC > 16 mg/L) since no breakpoints for disk diffusion method are available in the EUCAST guidelines. Thirteen antibiotics were tested for *K. pneumoniae* isolates following EUCAST standards (2022): ticarcillin-clavulanic acid (75–10 μg), cefoxitin (30 μg), ceftazidime (10 μg), cefepime (30 μg), aztreonam (30 μg), imipenem (10 μg), meropenem (10 μg), ertapenem (10 μg), amikacin (30 μg), gentamicin (10 μg), ciprofloxacin (5 μg), trimethoprim–sulfamethoxazole (1.25/23.75 μg), and chloramphenicol (30 μg). Three antibiotics were tested for *K. pneumoniae* isolates following CLSI standards (2021) [18]: cefotaxime (30 μg), nalidixic acid (30 μg), and tetracycline (30 μg).

### 2.3. Determination of Minimum Inhibitory Concentration

The microdilution method was used to determine the minimum inhibitory concentration (MIC) of linear ε-PL (~4700 g/mol; Handary, Brussels, Belgium). Briefly, each isolate was seeded on Brain Heart Infusion (BHI) agar (Frilabo, Maia, Portugal) and incubated at 37 °C for 24 h. Then, the bacterial cells were subcultured in tubes containing Mueller-Hinton (MH) broth (Frilabo, Maia, Portugal) and incubated overnight at 37 °C with 150 rpm in the ES-20/60 Orbital Shaker-Incubator (Biosan, Riga, Latvia). The overnight culture was diluted in fresh MH broth to a turbidity standard of 0.5 McFarland using a spectrophotometer. Then, Epsilon-poly-L-lysine was diluted in sterilized distilled water at different concentrations (0 µg/mL to 8182 µg/mL), and 75 µL of each concentration was placed in a polystyrene flat bottom 96-well plate. Also, 75 µL of bacterial suspension was placed in the same 96-well microtiter plate and incubated for 24 h at 37 °C with 150 rpm. Bacterial growth was determined at 630 nm using a microplate reader, the BioTek ELx808U (BioTek, Winooski, VT, USA). MIC was defined as the lowest concentration that prevents bacterial growth.

### 2.4. Biofilm Formation and Biomass Quantification

The bacterial adhesion of all isolates was assessed using a microtitre plate-based assay as previously described with some modifications [19]. Briefly, to perform the assay, one colony from each bacterial culture that had grown overnight on brain heart infusion (BHI) agar was suspended in Luria-Bertani (LB) broth (Oxoid^®^, Basingstoke, UK) and incubated for 24 h at 37 °C. Then, the bacterial suspension was diluted to 0.5 on the McFarland scale using Tryptic Soy Broth (TSB) (Oxoid^®^, Basingstoke, UK). Then, 100 µL of each dilution was added to each well of a 96-well flat-bottom microplate. *Pseudomonas aeruginosa* ATCC^®^ 27853 was included in all microplates as a positive control, and TSB without bacterial inoculum was used as a negative control. The microplates were incubated for 24 h at 37 °C. For quantitative assays, eight replicate wells were used for each treatment. After incubation, bacterial cells in suspension were removed by turning the microplates over, and they were washed twice with distilled water. This step helps remove stray cells and media components that may be stained in the next step, significantly reducing background staining. Subsequently, 125 µL of a 0.1% (*v*/*v*) solution of crystal violet (CV) (Frilabo, Maia, Portugal) was introduced into each well of the microplate, followed by an incubation period at room temperature for 10–15 min. Post-incubation, the CV solution was decanted, and the microplates underwent 3–4 washes with distilled water. Following this, meticulous drying of the plates was conducted using paper towels to eliminate any residual cells and stains, after which they were left to air-dry overnight.

To determine the biomass of the biofilm, 125 µL of 30% (*v*/*v*) acetic acid was added to each well of the microplate to dissolve the crystal violet. After an additional incubation period at room temperature for 10–15 min, optical density readings were taken at 630 nm (OD630nm) using a microplate reader (BioTek ELx808U, BioTek, Winooski, VT, USA). An uninoculated well containing 30% acetic acid served as a reference blank.

Following the MIC results, the same protocol was utilized for the production of biofilms, namely with the use of crystal violet, with one exception. Instead of 125 µL of 0.1% (*v*/*v*) CV solution, we added 175 µL since each well once contained 150 µL of liquid (75 µL of bacterial suspension and 75 µL of ε-PL solution), and we wanted the CV solution to cover all the biofilm that had eventually formed. The subsequent steps, including incubation, washing, drying, and biomass quantification, adhered to the protocol. Optical density was measured using a microplate reader (BioTek ELx808U, Lonza’s Vision Zero), with an uninoculated well containing 30% acetic acid serving as the blank control.

### 2.5. Antimicrobial Resistance and Virulence Genes

All isolates were studied for the presence of antimicrobial resistance genes according to the phenotypic results. DNA extraction was performed by following the boil method protocol [20]. Briefly, three colonies of overnight-growth bacteria were placed in a test tube containing 500 µL of distilled water and boiled for 8 min in a water bath. After, the tubes were vortexed vigorously and centrifuged for 2 min at 12,000 rpm. Then, 490 µL of the supernatant was recovered. Afterward, DNA concentration was determined using a NanoDrop system, and polymerase chain reaction (PCR) was performed. For *P. aeruginosa* isolates, the following resistance genes were tested: *bla*_TEM_, *bla*_SHV_, *bla*_CTX_, *bla*_PER_, *bla*_SME_, *bla*_KPC_, *bla*_Gim_, *bla*_Smp_, *bla*_Vim_, *bla*_Vim-2_, *bla*_NDM_, *bla*_OXA_, *acc*(3)-*I*, *aac*(3)-*II*, *aac*(3)-*III*, *aac*(3)-*IV*, and *ant*(2′)-*Ia*. For *K. pneumoniae* isolates, the following resistance genes were tested: *bla*_TEM_, *bla*_SHV_, *bla*_CTX_, *bla*_KPC_, *bla*_NDM_, *bla*_OXA_, *acc*(3)-*II*, *aac*(3)-*IV*, *tetA*, *tetB*, *cmlA*, *catA*, *sul1*, *sul2*, *sul3*, and *dfrA* [21,22,23,24,25,26,27,28,29,30,31,32,33,34,35,36,37,38,39].

### 2.6. Multilocus Sequence Typing

Multilocus sequence typing (MLST) for selected *P. aeruginosa* and *K. pneumoniae* isolates was performed by PCR and sequencing of seven housekeeping genes (*acsA*, *aroE*, *guaA*, *mutL*, *nuoD*, *ppsA*, and *trpE* for *P. aeruginosa*; *gapA*, *infB*, *mdh*, *pgi*, *phoE*, *rpoB*, and *tonB* for *K. pneumoniae*). Allelic profiles and sequence types (STs) were compared with the PubMLST and BIGSdb-Pasteur databases (http://pubmlst.org/paeruginosa/; https://bigsdb.pasteur.fr/klebsiella/, accessed on 28 April 2023).

## 3. Results and Discussion

### 3.1. Antimicrobial Susceptibility Testing

All phenotypic profiles of each *P. aeruginosa* and *K. pneumoniae* isolate are indicated in Table 1. *P. aeruginosa* isolates included in this study showed, in most cases, resistance to imipenem (85.7%). All *P. aeruginosa* isolates were susceptible to amikacin and colistin. On the other hand, ESBL-producing *K. pneumoniae* isolates showed resistance to cefotaxime (100%), cefepime (100%), and aztreonam (100%). Moreover, one of the *K. pneumoniae* isolates showed phenotypic resistance to imipenem (4.2%), and five additional isolates showed resistance to other carbapenems, such as ertapenem and/or meropenem (20.8%).

### 3.2. Antimicrobial Resistance Genes

After PCR analysis, some β-lactamase-related genes were detected in *P. aeruginosa* isolates, such as *bla*_SPM_ (35.7% of isolates), *bla*_KPC_ (35.7%), *bla*_SHV_ (14.3%), *bla*_CTX-M_ (14.3%), *bla*_OXA_ (14.3%), *bla*_TEM_ (7.1%), *bla*_PER_ (7.1%), *bla*_VIM_ (7.1%), and *bla*_VIM-2_ (7.1%) (Table 1). Multiple studies also report the presence of these genes in clinical *P. aeruginosa* isolates. Recently, in 2020, Ahmed et al. reported that 51% of *P. aeruginosa* from cancer patients were positive for the carbapenemase genes encoding VIM, GIM, and IMP enzymes, 38% for SPM and AIM, 30% for BIC, 20% for NDM and TEM, 17% for KPC, and 15% for OXA [40]. The first report of *bla*_VIM-2_ and *bla*_PER-1_ in Latin America was published in 2018 in a *P. aeruginosa* strain recovered from a cerebrospinal fluid sample [41]. The emergence of these β-lactamase genes in *P. aeruginosa* is a major concern in treating infections caused by this bacterium since they are associated with various resistance mechanisms to β-lactam antibiotics. The resistance to aminoglycosides expressed by two isolates was associated with the *aac*(3)-*IV* gene. AAC enzymes are commonly detected in aminoglycoside-resistant strains, as well as APH enzymes. Like the current study, Holbrook and Garneau-Tsodikova have also detected the *aac*(3)-*IV* gene in 10.7% of the isolates, together with *aph*(3′)-*Ia* (70.5%), *aac*(6′)-*Ib* (41.0%), and *ant*(2″)-*Ia* (5.7%) [42].

Since all *K. pneumoniae* isolates were ESBL-producers, most of the isolates carried, as expected, ESBL-related genes (Table 1). The most prevalent β-lactamase genes detected in our study were *bla*_CTX-M_ (91.7%) and *bla*_SHV_ (91.7%), followed by *bla*_TEM_ (83.3%). All the *bla*_CTX-M_ genes confer an ESBL phenotype, although only some variants of *bla*_TEM_ or *bla*_SHV_ are related to the ESBL phenotype. All *K. pneumoniae* have an intrinsic SHV resistance gene. However, in some cases, it could be mutated to an ESBL variant, or the isolate could acquire a new ESBL-SHV variant that can only be known by sequencing the obtained amplicons. The presence of these β-lactamases in *K. pneumoniae* continues to be reported worldwide. Multiple studies keep detecting these genes and their variants [43,44,45]. Moreover, the *bla*_KPC_ (16.7%) and *bla*_NDM_ (12.5%) carbapenemase genes were also found among our isolates. The emergence and spread of carbapenemase genes among clinical isolates is a significant concern since carbapenem antibiotics are often used as a last resort for treating infections caused by multidrug-resistant bacteria. The most common carbapenemase genes detected in *K. pneumoniae* are from the KPC [46,47], NDM [48,49], and OXA-48 families [50,51]. Different β-lactamase gene combinations were observed among our isolates, but the most predominant was *bla*_CTX-M_ + *bla*_TEM_ + *bla*_SHV_ (*n* = 12). Also, one *K. pneumoniae* strain (HS79) showed a combination of five different β-lactamase genes (*bla*_CTX-M_ + *bla*_TEM_ + *bla*_KPC_ + *bla*_OXA_ + *bla*_NDM_). In only one strain (HS50), no carbapenemase genes were detected, besides this strain being resistant to ertapenem. However, other carbapenemase genes have also been reported in *K. pneumoniae*, such as VIM [52,53], IMP [54,55], and GES [56,57], that may be conferring resistance to ertapenem in this isolate. In relation to resistance to sulfonamides and trimethoprim, we detected *sul2* (87.5%), *sul1* (41.7%), *sul3* (12.5%), and *dfrA* (8.3%) genes among the studied isolates. Like our study, Mbelle et al. also reported high rates of prevalence of *sul2* (86%) and *sul1* (78%) [58]. The *sul3* and *dfrA* genes are also commonly found in *K. pneumoniae*. Carvalho et al. [59] reported, in 2021, the presence of *sul2* in 10 *K. pneumoniae* clinical isolates and *sul3* in 5 isolates, but no *dfrA* was detected. However, the presence of *dfrA1* in four isolates and *dfrA17* in one isolate was reported by Yu et al. [60] after whole-genome sequencing of seven *K. pneumoniae* strains. Relatively to aminoglycoside resistance, we verified the presence of *aac*(3)-*II* (75%) and *aadA1* (62.5%). The same was reported by Lomonaco et al. [43], who detected the presence of these two genes in ten MDR clinical isolates of *K. pneumoniae* from the United States of America. Moreover, *tetA* (12.5%) was the only gene detected that confers resistance to tetracyclines. The absence of *tetA* or *tetB* genes was observed in certain tetracycline-resistant isolates. This could be attributed to several factors, such as the presence of alternative tetracycline resistance genes like *tetC*, *tetD*, or *tetG* [61] or the emergence of tetracycline resistance through mutations in bacterial ribosomes or other cellular components independent of specific resistance genes [62]. The resistance to chloramphenicol was proven by the presence of the *cmlA* gene (37.5%) (Table 1). The *cmlA* gene is a common mechanism of resistance to chloramphenicol. It is often located on mobile genetic elements such as plasmids, which can facilitate its spread between bacterial species and contribute to the emergence of multidrug-resistant strains. *cmlA*-mediated resistance to chloramphenicol has been reported in various bacterial species, including *K. pneumoniae* [53].

### 3.3. Multilocus Sequence Typing

After the biofilm production results, we selected seven *K. pneumoniae* and seven *P. aeruginosa* isolates for sequence typing. The results are presented in Table 2.

These sequence types (STs) are commonly found in *K. pneumoniae* strains: ST15, ST307, and ST348 are known to be widespread and have been associated with hospital-acquired infections [63,64,65], while ST584 is a relatively new ST that has been identified in *K. pneumoniae* strains from clinical and environmental sources [66,67]. The STs found in *P. aeruginosa* have been associated with hospital-acquired infections: ST285, ST274, ST1404, and ST228 have been identified in hospitals in several European countries. The other STs have been reported in hospitals in Brazil, China, the United States, and Saudi Arabia [68]. Interestingly, we found two new allelic combinations for HU5 and HU6 isolates. HU5 seems to be a single locus variant (SLV) of ST1228 with a different *trpE* allele. HU6 seems to be an SLV of ST170/ST997/ST1315/ST2454 with a different *mutL* allele. The identification of new allelic combinations in *P. aeruginosa* is not uncommon, as this bacterium has a high level of genetic diversity [69]. SLVs are typically defined as strains that differ from each other by a single nucleotide polymorphism (SNP) in one of the seven MLST loci, and they are often considered to be related to each other in an evolutionary sense.

### 3.4. Determination of Minimum Inhibitory Concentration (MIC) for Epsilon-poly-L-lysine

**Figure 1:**
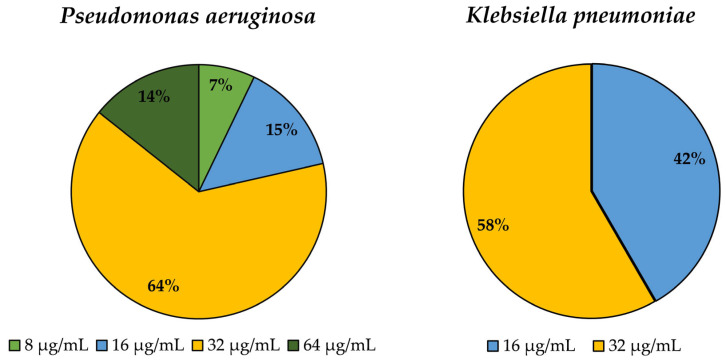
Determination of minimum inhibitory concentration for Epsilon-poly-L-lysine against *P. aeruginosa* (Pa) and *K. pneumoniae* (Kp) isolates.

*In vitro* activity of ε-PL against the *P. aeruginosa* and *K. pneumoniae* isolates showed relatively high MICs. MIC values ranged between 8 and 64 µg/mL in *P. aeruginosa* isolates, with 64.3% having a MIC value of 32 µg/mL and between 16 and 32 µg/mL in *K. pneumoniae* isolates, with 58.3% also having a MIC value of 32 µg/mL (Figure 1). The high MIC values observed for ε-PL against the tested *P. aeruginosa* and *K. pneumoniae* isolates suggest that these bacteria have relatively low susceptibility to the antimicrobial effects of ε-PL. The results from the electron microscopy analysis of *E. coli* O157:H7 cells, reported by Zhang et al. [70], suggest that ε-PL can cause damage to the bacterial cell wall or membrane even at concentrations lower (16 µg/mL) than those observed in our study. This supports the idea that the relatively high MICs observed in the current study may be due to factors such as the limited permeability of the bacterial cell wall or membrane to ε-PL rather than the inability of ε-PL to exert its antimicrobial effects once inside the bacterial cell. However, further studies would be needed to confirm this hypothesis. Moreover, the activity should be tested in the future under different methodological conditions (media and bacteria density, among others) to check if these variables could affect the activity of the agent. In another recent study, it was observed that ε-PL caused alterations in the morphology of *P. aeruginosa* cells, resulting in protrusions on the cell wall, as evidenced by scanning electron microscopy [71]. Additionally, ε-PL demonstrated the ability to depolarize and permeabilize the bacterial membrane over time, which may enhance the effectiveness of antibiotics. The MIC in the referred study turned out to be higher, around 50 µg/mL, using the reference strain PAO1. Experiments also evaluated the efficacy of ε-PL in combination with imipenem against both sensitive and antibiotic-resistant strains of *P. aeruginosa*. A synergy between ε-PL and imipenem was observed, resulting in a significant reduction in bacterial growth, especially against resistant strains. These findings suggest that ε-PL could be a promising option for treating *P. aeruginosa* infections, particularly those resistant to conventional antibiotics. The ability of ε-PL to increase bacterial membrane permeability may enhance the effectiveness of antibiotics, making it a potential therapeutic adjuvant against resistant bacterial infections. Regarding *K. pneumoniae*, this field of research remains an enigma, thereby underscoring the significance of this study.

### 3.5. Biofilm Formation and Biomass Quantification

**Figure 2:**
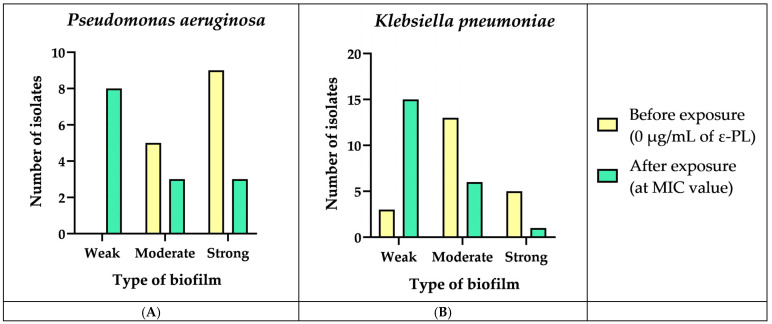
Effect of the ε-PL on the biofilm formation against 14 *P. aeruginosa* and 24 *K. pneumoniae* isolates. (**A**) Before exposure to ε-PL, the 14 *P. aeruginosa* isolates were strong and moderate biofilm producers. After exposure to ε-PL at the MIC value, a reduction in biofilm production was verified in 11 isolates. The number of strong and moderate biofilm producers’ isolates has reduced, and the number of weak biofilm producers has increased. (**B**) Before exposure to ε-PL, 21 *K. pneumoniae* isolates were strong, moderate, and weak biofilm producers. After exposure to ε-PL at the MIC value, a reduction in biofilm production was verified in 16 isolates. The number of strong and moderate biofilm producers’ isolates has reduced, and the number of weak biofilm producers has increased.

We verified that all *P. aeruginosa* isolates (*n* = 14) were biofilm producers (100%), of which five were moderate producers (35.7%) and nine were strong producers (64.3%) (Figure 2A). Among *K. pneumoniae* isolates, we verified that 21 of them were biofilm producers (87.5%), of which 5 were strong producers (20.8%), 13 were moderate producers (54.2%), and 3 were weak producers (12.5%) (Figure 2B). After the exposure to ε-PL, we verified a decrease in biofilm production in 11 *P. aeruginosa* and 16 *K. pneumoniae* strains, i.e., in these strains, we verified that the concentration of ε-PL was inversely proportional to the production of biofilm, which means that the strains that were initially strong/moderate biofilm producers at MIC values became weak/null biofilm producers (Figure 2). Several studies have investigated the effects of ε-PL on biofilm production in different bacterial species. Some studies have shown similar results to the current study. For example, a study conducted on *Salmonella typhimurium* showed that ε-PL inhibits biofilm formation in a dose-dependent manner. Additionally, ε-PL at low concentrations did not affect the viability of bacterial cells. This suggests that ε-PL may have a selective effect on biofilms, inhibiting their formation while leaving bacterial cells intact [72]. Another study investigated the effect of ε-PL on the cell structure and biofilm formation in *Cronobacter sakazakii*. The results showed that ε-PL had a MIC of 256 μg/mL against *C. sakazakii*. ε-PL was found to reduce the surface hydrophobicity and motility of *C. sakazakii*, which affects the formation of biofilm. ε-PL also demonstrated a significant effect on the inhibition and eradication of biofilm, and the removal efficiency of biofilm was significantly improved when combined with physical oscillation. This means that ε-PL destroyed the cell structure of *C. sakazakii*, and the bacteriostatic effect was achieved while inhibiting biofilm formation and removing mature biofilm [73]. Overall, these studies indicate that ε-PL may serve as a natural antimicrobial agent that can hinder the formation of biofilms in diverse bacterial species. However, further research is needed to determine the optimal concentration and application methods for different bacterial species and to investigate the potential mechanisms of action of ε-PL on biofilm production. Surprisingly, one of the *K. pneumoniae* isolates (HS55) showed an opposite relationship between ε-PL concentration and biofilm production, moving from non-producer to weak biofilm producer at 4 µg/mL of ε-PL. It is not uncommon for some bacteria to display different responses to antibacterial agents due to variations in their genetic background or environmental conditions. For instance, a study reported that low concentrations of triclosan, an antimicrobial agent, can promote the binding of *S. aureus* to inanimate surfaces such as plastic and glass [74].

Another type of consideration that must be made is related to the potential of ε-PL in a clinical setting. ε-PL, with its antimicrobial properties against Gram-negative bacteria like *P. aeruginosa* and *K. pneumoniae*, holds significant promise as a potential therapeutic agent. In a clinical setting, ε-PL might be considered for topical application, where ε-PL could be formulated into creams, ointments, or gels for the treatment of localized skin infections or wounds [75,76]. Additionally, ε-PL could be utilized in medical devices such as catheters or wound dressings to prevent bacterial colonization and biofilm formation [72].

Furthermore, ε-PL could be explored in the future for the treatment of systemic infections caused by MDR bacteria. However, further research would be needed to determine the potential side effects associated with systemic ε-PL administration, as well as the optimal dosage and administration route. Overall, the successful translation of ε-PL from preclinical studies to clinical applications would require rigorous evaluation of its safety, efficacy, and pharmacokinetic properties in human trials.

## 4. Conclusions

In this study it was performed the genetic characterization of β-lactamase genes in the collection of *P. aeruginosa* and *K. pneumoniae* clinical isolates used as target for testing the activity of ε-PL agent; it has been shown the carriage of a wide range of relevant β-lactamases, including those related to ESBL and carbapenemases, according to the phenotypes of the selected isolates.

On the other hand, this study found high MICs in isolates of both bacterial species for ε-PL, with the majority of *P. aeruginosa* and *K. pneumoniae* isolates having a MIC value of 32 µg/mL. These MIC values suggest that these bacteria require relatively high concentration of ε-PL compound to be inhibited under the studied conditions, emphasizing the need for preliminary screening and/or optimization of the polymer, considering the specificity of the intended application. Despite these values, it is important to note that this natural polymer is highly customizable, demonstrating broad efficacy, low toxicity, and a low probability of leading to the development of resistant strains. In the context of this work and considering these properties, new multidisciplinary research efforts are prompted to enhance its efficacy against *P. aeruginosa* and *K. pneumoniae* and to leverage its clinical application.

## Figures and Tables

**Table 1:** Phenotypic and genotypic characterization of resistance to different antibiotics in the selected *P. aeruginosa* (Pa) and *K. pneumoniae* (Kp) isolates.

Species	Isolate	MDR	Phenotype of Resistance	Resistance Genes
Pa	HU1	−	IMI, MEM, LEV	*bla*_KPC_
	HU2	−	IMI	*bla*_KPC_
	HU4	+	PTZ, CAZ, ATM, IMI, MEM, DOR	*bla*_KPC_
	HU5	−	IMI, MEM, LEV	*bla*_SPM_
	HU6	−	IMI	*bla*_KPC_, *bla*_SPM_
	HU7	−	IMI	*bla*_SPM_
	HU8	+	PTZ, TTC, CAZ, ATM, CIP,	*bla*_CTX-M_, *bla*_VIM2_, *bla*_SPM_
	HU9	−	IMI	*bla*_SHV_
	HU10	−	IMI	*bla*_SHV_, *bla*_VIM_
	HU11	−	IMI	*bla*_TEM_, *bla*_SPM_
	HU12	−	IMI	*bla*_CTX-M_, *bla*_OXA_
	HU13	−	TOB, CN	*aac*(3)-*IV*
	HU14	−	IMI, CN	*bla*_PER_, *bla*_OXA_, *aac*(3)-*IV*
	HU15	−	IMI	*bla*_KPC_
Kp	HS2	+	TTC, CAZ, CTX, FEP, ATM, CN, TET, CIP, SXT	*bla*_CTX-M_, *aac*(3)-*II*, *aadA1*, *sul2*
	HS8	+	TTC, FOX, CAZ, CTX, FEP, ATM, CN, TET, CIP, NA, SXT	*bla*_CTX-M_, *bla*_TEM_, *bla*_SHV_, *aac*(3)-*II*, *sul2*
	HS14	+	TTC, CAZ, CTX, FEP, ATM, AK, CIP, NA, SXT	*bla*_CTX-M_, *bla*_TEM_, *bla*_SHV_, *aac*(3)-*II*, *sul2*
	HS17	+	TTC, FOX, CAZ, CTX, FEP, ATM, CN, CIP, NA, SXT, CHL	*bla*_CTX-M_, *bla*_TEM_, *bla*_SHV_, *aac*(3)-*II*, *aadA1*, *cmlA*, *sul2*
	HS29	+	TTC, CAZ, CTX, FEP, ATM, TET, CIP, SXT	*bla*_CTX-M_, *bla*_TEM_, *bla*_SHV_, *tetA*, *sul2*
	HS30	+	TTC, FOX, CAZ, CTX, FEP, ATM, MEM, ERT, IMI, AK, CN, CIP, SXT	*bla*_TEM_, *bla*_SHV_, *bla*_KPC_, *aac*(3)-*II*, *aadA1*, *sul2*
	HS34	+	TTC, CAZ, CTX, FEP, ATM, CN, CIP, NA, SXT, CHL	*bla*_CTX-M_, *bla*_SHV_, *aac*(3)-*II*, *aadA1*, *cmlA*, *sul2*
	HS36	+	TTC, FOX, CAZ, CTX, FEP, ATM, MEM, ERT, AK, CN, CIP, NA, SXT	*bla*_CTX-M_, *bla*_TEM_, *bla*_SHV_, *bla*_KPC_, *aac*(3)-*II*, *aadA1*, *sul2*
	HS42	+	TTC, CAZ, CTX, FEP, ATM, CN, CIP, NA, SXT	*bla*_CTX-M_, *bla*_TEM_, *bla*_SHV_, *aac*(3)-*II*, *aadA1*, *sul2*, *sul1*
	HS50	+	TTC, CAZ, CTX, FEP, ATM, ERT, AK, CN, SXT	*bla*_CTX-M_, *bla*_TEM_, *bla*_SHV_, *aac*(3)-*II*, *aadA1*, *sul1*, *sul2*
	HS54	+	TTC, CAZ, CTX, FEP, ATM, ERT, CIP, SXT	*bla_TEM_*, *bla*_SHV_, *bla*_NDM_, *sul2*, *sul1*
	HS55	+	TTC, CAZ, CTX, FEP, ATM, CN, CIP, NA, SXT, CHL	*bla*_CTX-M_, *bla*_TEM_, *bla*_SHV_, *aac*(3)-*II*, *aadA1*, *cmlA*, *sul2*
	HS56	+	TTC, FOX, CAZ, CTX, FEP, ATM, MEM, ERT, AK, CN, CIP, NA, CHL	*bla*_CTX-M_, *bla*_SHV_, *bla*_KPC_, *bla*_NDM_, *aac*(3)-*II*, *aadA1*, *cmlA*
	HS57	+	TTC, CAZ, CTX, FEP, ATM, TET, CIP, SXT	*bla*_CTX-M_, *bla*_TEM_, *bla*_SHV_, *tetA*, *sul2*
	HS58	+	TTC, CAZ, CTX, FEP, ATM, CN, TET, CIP, NA, SXT, CHL	*bla*_CTX-M_, *bla*_SHV_, *aac*(3)-*II*, *cmlA*, *sul1*
	HS67	+	CAZ, CTX, FEP, ATM, CN, CIP, SXT	*bla*_CTX-M_, *bla*_TEM_, *bla*_SHV_, *aac*(3)-*II*, *aadA1*, *sul2*
	HS77	+	TTC, CAZ, CTX, FEP, ATM, CN, CIP, NA, SXT, CHL	*bla*_CTX-M_, *bla*_TEM_, *bla*_SHV_, *aac*(3)-*II*, *aadA1*, *sul1*, *sul2*, *sul3*
	HS79	+	TTC, FOX, CAZ, CTX, FEP, ATM, ERT, CIP	*bla*_CTX-M_, *bla_TEM_*, *bla*_KPC_, *bla*_OXA_, *bla*_NDM_
	HS81	+	TTC, CAZ, CTX, FEP, ATM, CN, CIP, NA, SXT, CHL	*bla*_CTX-M_, *bla*_TEM_, *bla*_SHV_, *aac*(3)-*II*, *aadA1*, *cmlA*, *sul1*, *sul2*, *sul3*
	HS84	+	TTC, CAZ, CTX, FEP, ATM, CN, CIP, NA, SXT, CHL	*bla*_CTX-M_, *bla*_TEM_, *bla*_SHV_, *aac*(3)-*II*, *aadA1*, *cmlA*, *sul1*, *sul2*, *dfrA*
	HS87	+	CTX, FEP, ATM, CIP, NA, SXT	*bla*_CTX-M_, *bla*_TEM_, *bla*_SHV_, *sul1*, *sul2*, *dfrA*
	HS91	+	TTC, CAZ, CTX, FEP, ATM, CIP, NA, SXT	*bla*_CTX-M_, *bla*_TEM_, *bla*_SHV_, *sul1*, *sul2*
	HS92	+	TTC, CAZ, CTX, FEP, ATM, AK, CIP, NA, SXT	*bla*_CTX-M_, *bla*_TEM_, *bla*_SHV_, *aac*(3)-*II*, *aadA1*, *sul1*, *sul2*
	HS101	+	CAZ, CTX, FEP, ATM, CN, TET, CIP, SXT, CHL	*bla*_CTX-M_, *bla*_TEM_, *bla*_SHV_, *aac*(3)-*II*, *aadA1*, *tetA*, *cmlA*, *sul1*, *sul2*

TTC—ticarcillin-clavulanate; FOX—cefoxitin; CAZ—ceftazidime; CTX—cefotaxime; FEP—cefepime; ATM—aztreonam; MEM—meropenem; ERT—ertapenem; IMI—imipenem; AK—amikacin; CN—gentamicin; TET—tetracycline; CIP—ciprofloxacin; NA—nalidixic acid; SXT—trimethoprim-sulfamethoxazole; CHL—chloramphenicol; DOR—doripenem; PTZ—piperacillin; TOB—tobramycin.

**Table 2:** MLST types of seven clinical *P. aeruginosa* isolates and seven clinical *K. pneumoniae* isolates.

*P. aeruginosa*		*K. pneumoniae*	
Isolate	ST	Isolate	ST
HU2	699	HS8	307
HU5	X *	HS29	348
HU6	X *	HS34	15
HU7	1338	HS36	307
HU10	285	HS50	584
HU14	274	HS55	15
HU15	1404	HS58	307

* New allelic combination.

## Data Availability

Data are contained within the article.
